# Atmospheric Pressure Plasma Processing of an Optical Sinusoidal Grid

**DOI:** 10.3390/mi10120828

**Published:** 2019-11-28

**Authors:** Duo Li, Na Li, Xing Su, Peng Ji, Bo Wang

**Affiliations:** Center for Precision Engineering, Harbin Institute of Technology, Harbin 150001, China

**Keywords:** sinusoidal grid, atmospheric pressure plasma processing, post-polishing

## Abstract

Sinusoidal grid with nanometric precision is adopted as a surface encoder to measure multiple degree-of-freedom motions. This paper proposes the atmospheric pressure plasma processing (APPP) technique to fabricate an optical sinusoidal grid surface. The characteristics of removal function and surface generation mechanism are firstly presented. Both simulation and experiment validate the effectiveness of APPP to fabricate a sinusoidal grid surface with nanometric precision. Post mechanical polishing experiments show that APPP features can be well maintained while the surface roughness is greatly reduced to meet the optical requirement.

## 1. Introduction

**Figure 1:**
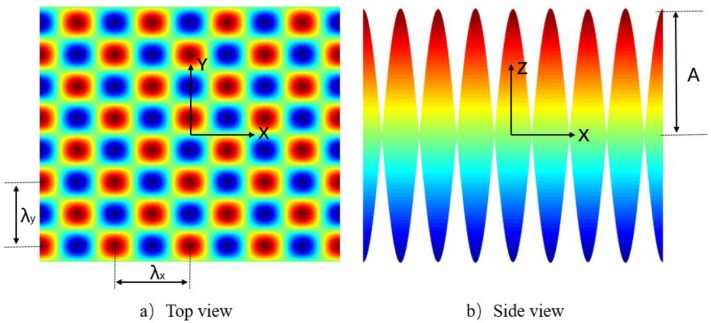
Schematic of sinusoidal grid surface.

As an innovative surface encoder, a sinusoidal grid has been successfully adopted to measure multiple degree-of-freedom translational and tilt motions of precision stages [1,2,3]. As shown in Figure 1, a sinusoidal grid is superposition of two-dimensional sinusoidal curves in the X direction and the Y direction, respectively. The surface can be described as a mathematical function,

(1) $$z=A\cos(\frac{2\pi }{\lambda_{x}}x+\varphi_{x})\sin(\frac{2\pi }{\lambda_{y}}y+\varphi_{y})+B$$

where *A* is amplitude, *B* is the offset and *λ_x_*, *λ_y_* are wavelengths in the two orthogonal X–Y dimension. *φ_x_* and *φ_y_* are the phase angles to adjust the lateral offset of the sinusoidal structures.

As the translational reference of a multiple degree-of-freedom encoder, the sinusoidal grid is preferred with form accuracy in nanometric level, in order to guarantee the encoder functional performance. Single-point diamond turning (SPDT) with tool servo technique is widely adopted to fabricate sinusoidal grid surfaces. For example, Gao et al. [4] developed a fast tool servo (FTS) unit to fabricate large area sinusoidal structures with 100 μm wavelength and 100 nm amplitude. A center alignment method and discrete Fourier transform analysis were also used to evaluate the machining accuracy. Zhang et al. [5] proposed a cylindrical coordinate method with optimized tool geometry for slow tool servo turning. The cutting process was simulated beforehand and a sinusoidal surface was fabricated with 5.54 nm Ra. Ji et al. [6] fabricated a compound sinusoidal grid surface, which was added on a paraboloid basis. The tool path was generated by the combination of constant angle and constant arc-length methods. Form accuracy of 4.25 µm (PV) and surface roughness of 89 nm (Ra) were achieved. However, the SPDT technique is limited to certain machinable materials, tool wear and machining efficiency [7]. In addition, it is difficult to achieve form accuracy at a nanometric level.

Optical materials, such as fused silica, are highly preferred in opto-electronics systems due to their excellent optical, chemical and mechanical properties. Optical sinusoidal grid surfaces can be used in the next generation of multiple degree-of-freedom encoders. Atmospheric pressure plasma processing (APPP) is a promising technique in optical fabrication because of its deterministic high material removal rate and non-contact removal mechanism. It is based on a pure chemical reaction between silicon-based materials and reactive fluorine radicals generated by the plasma at atmospheric pressure, which avoids mechanical damage and decreases the processing cost. Takino et al. [8] studied the removal characteristics of chemical vaporization machining (CVM) with radio frequency plasma with a pipe electrode. The results showed that the removal rate of plasma CVM could be equal to that of precision grinding, while the roughness of the processed surfaces was the same as that of the polished surfaces. Arnold et al. [9] proposed the atmospheric plasma jet machining-based manufacturing chain for freeform optics. A complex finite element (FE) heat transfer model was built to compensate spatio-temporal variation issues. The figuring efficiency was increased by an iterative correction of the targeted removal according to modelling results [10]. Li et al. [11] established a multi-physics model for the APPP torch, including discharge, fluid field and surface chemical reactions. It was found that the distribution of active F atoms was Gaussian and the ratio of O/CF*_x_* affected the surface morphology formation. The existence of cellular microstructures caused an opacification phenomenon on the silica optics [12]. Jourdain et al. [13] adopted an inductively coupled plasma (ICP) torch with De-Laval nozzle to Figure large fused silica optics. An adapted tool-path was applied to reduce the thermal effect of a plasma torch for better convergence.

The controllable Gaussian-shape removal function makes APPP potential to generate structured and freeform surfaces with nanometric form accuracy and high efficiency. However, little detailed research has been reported on the fabrication of optical sinusoidal grid surface with APPP and post processing issues. In this paper, APPP technique is proposed to fabricate optical sinusoidal grid surface (fused silica material). The APPP platform is firstly presented and its removal function is analyzed. Then, a surface generation mechanism is elaborated and simulation analysis is conducted to verify the dwell time solution. Finally, the experimental APPP with post mechanical polishing is carried out to validate the effectiveness to fabricate an optical sinusoidal grid surface.

## 2. APPP Platform and Surface Generation Principle

### 2.1. Platform Configuration and Removal Function

**Figure 2:**
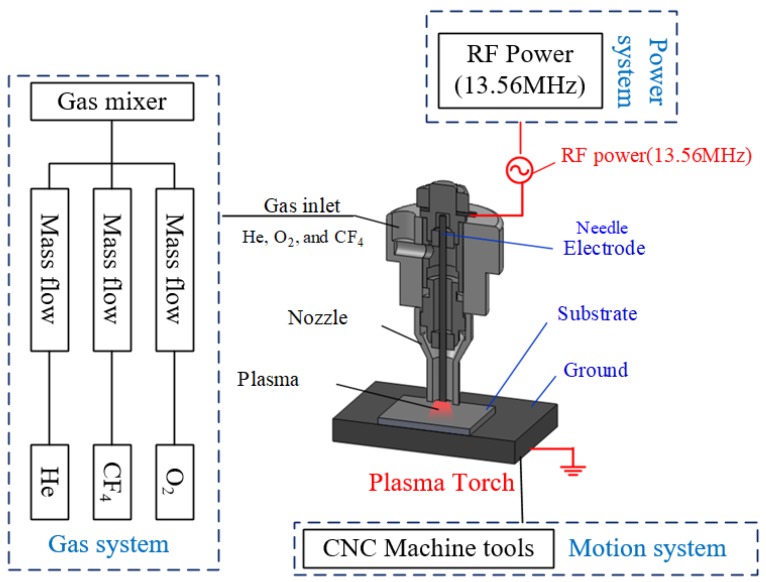
Schematic diagram of the atmospheric pressure plasma processing (APPP) system [11].

APPP platform comprises a capacitively coupled plasma generation system, a gas supply module, and multi-axis motion stages. A schematic diagram of the APPP platform is illustrated in Figure 2. A 13.56 MHz radio frequency power is applied to the electrode as a positive electrode and the workbench is grounded. The sample placed on the workbench serves as a dielectric barrier layer and the plasma is generated according to the principle of dielectric barrier discharge (DBD). The inner mixed gases, including He, O_2_, and CF_4_, are controlled by the multichannel mass flow controller. The plasma (He and O_2_) generated by radio frequency power serves as a chemical reactor and reactive radicals (F) are generated after the decomposition of CF_4_. The addition of O_2_ is used to improve the removal rate, as the unsaturated particles CF*_x_* produced by CF_4_ excitation are very likely to react with oxygen atoms and increase the concentration of active F atoms. Then, these reactive radicals (F) diffuse to the substrate and react with the sample surface material (SiO_2_). Because reaction products are volatile, the removal process is accomplished. APPP is considered as a pure chemical reaction because under atmospheric pressure, the maximum free path of the particles is small and the collision frequency is extremely high, which cannot form an accelerated bombardment effect. The balanced chemical reaction equation can be described as ${\mathrm{SiO}}_{2}+{\mathrm{CF}}_{4}\to {\mathrm{SiF}}_{4}↑+{\mathrm{CO}}_{2}↑$.

**Figure 3:**
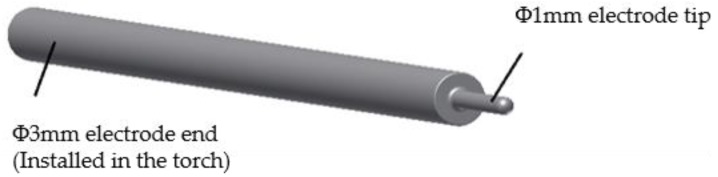
Schematic of needle electrode.

The electrode material is aluminum alloy (6061) and its size is changeable to meet different processing resolution requirements. In this work, the electrode tip is selected as 1 mm diameter, as shown in Figure 3.

**Figure 4:**
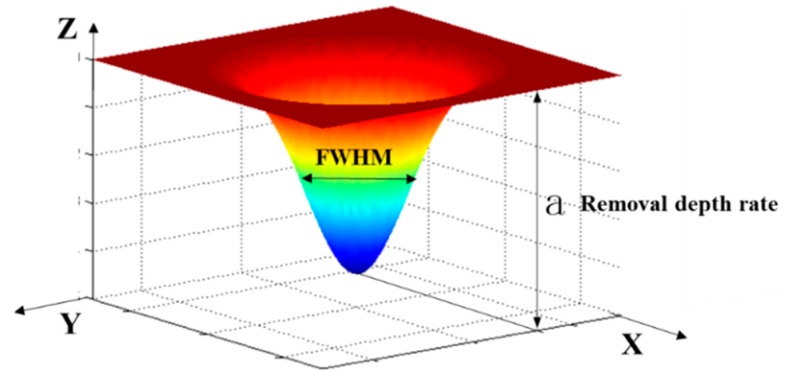
Gaussian shape removal function of APPP.

The investigation of removal function is essential for APPP, which is a computer-controlled optical surfacing (CCOS) method. As shown in Figure 4, the APPP removal function is typically of Gaussian shape, which is characterized by two parameters, removal depth rate and the full width at half maximum (FWHM).

The measured removal function can be fitted as follows,

(2) $$r\left(x,y\right)=a\cdot e^{-\frac{x^{2}+y^{2}}{2\sigma^{2}}}$$

where a is removal depth rate and σ is standard deviation of Gaussian function.

The full width at half maximum (FWHM), which determines the fabrication resolution in lateral scale, can be expressed as,

(3) $$\mathrm{FWHM}=2\sqrt{2\ln2}\sigma$$

In addition, the removal volume, which indicates processing efficiency, can be derived according to the Equations (2) and (3) as,

(4) $$V_{k}=\iint ae^{-\frac{x^{2}+y^{2}}{2\sigma^{2}}}dxdy=2a\sigma^{2}{\left(\int_{-\infty }^{+\infty }e^{-\theta^{2}}d\theta \right)}^{2}=2\pi a\sigma^{2}=\frac{\pi }{4\ln2}aFWHM^{2}$$

**Figure 5:**
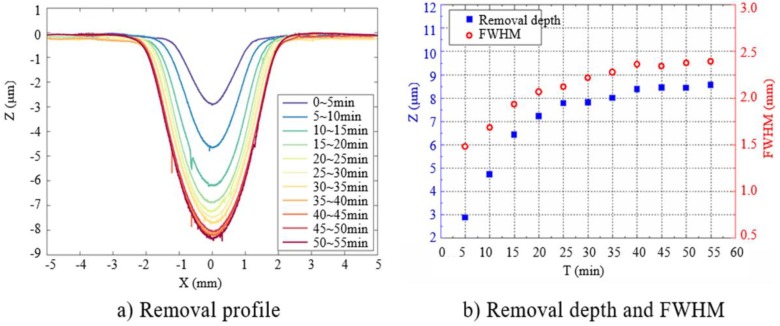
Profile measurement and analysis of removal function.

The removal function is experimentally obtained. The process parameters used are listed in Table 1. A single-point removal process (totally 11 points) was performed on an SiO_2_ sample. The dwell time of each point was equal to 5 min. The removal function of each point was measured by a stylus profilometer (PGI 1240, Taylor Hobson, Leicester, UK). The results are plotted in Figure 5.

It can be seen that the removal depth rate and FWHM increase gradually with time from 0 to 20 min. In the range of 20–40 min, the changes of the two are getting slower. After 40 min, the removal function tends to be stable. Therefore, it is necessary to stabilize the plasma source for more than 40 min before fabrication.

After the plasma stabilization process, repeatability experiments of removal function were performed using the same process parameters as shown in Table 1.

The results indicate that the maximum deviation of removal depth is about 5%, and the maximum deviation in FWHM is about 3.6%, which could meet the APPP requirements [14]. Therefore, using the parameters shown in Table 1, the stabilized removal function can be obtained with a removal depth rate 1.65 μm/min and FWHM 2.4 mm. The corresponding volume removal rate is 0.0108 mm^3^/min.

### 2.2. Surface Generation Principle for Sinusoidal Grid

**Figure 6:**
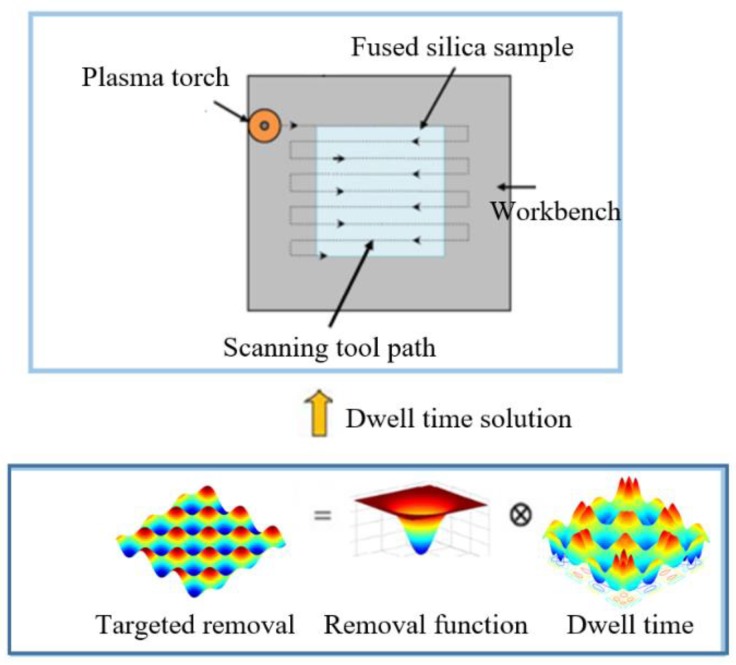
Schematic of APPP fabrication flow for sinusoidal grid.

As a sub-aperture CCOS method, the surface generation of APPP is based on the control of the dwell time of plasma torch on the sample surface according to the desired removal and removal function. In this work, the target removal is the reversal of designed sinusoidal grid surface data and removal function is obtained experimentally as shown in Section 2. Thus, in order to control the APPP to generate the sinusoidal grid surface, corresponding dwell time needs to be solved and transferred to the motion control. A schematic of APPP fabrication flow for sinusoidal grid surface is illustrated in Figure 6.

Mathematically, the target removal amount is the convolution (⊗) between the removal function and corresponding dwell time. It can be expressed by the mathematical model as shown in the following equation,

(5) F(x,y)=r(x,y)⊗T(x,y)

where *F*(*x*, *y*) is target removal, *r*(*x*, *y*) is removal function and *T*(*x*, *y*) is the corresponding dwell time. The linear equations method is used to calculate the dwell time with high efficiency and accuracy [15,16]. The convolution process is transformed into matrix multiplication. In Equation (5), n2-dimensional column vector *F* is the ideal removal amount of the designed surface shape. The ideal removal distribution is divided into n × n discrete points based on the processing step. *T* equals dwell time at discrete points. R is the removal function matrix. The element r*_ij_* in matrix *R* denotes the unit removal amount at point *i* when the plasma torch dwells at point *j*. Then, the Equation (5) can be converted into the matrix-based form as follows,

(6) $$\begin{array}{l} \;F\;=\;R\;\cdot \;T\\ \left[\begin{array}{c} f_{1}\\ f_{2}\\ \vdots \\ f_{n^{2}} \end{array}\right]=\left[\begin{array}{cccc} r_{11}^{\prime } & r_{12}^{\prime } & \cdots & r_{1n^{2}}^{\prime }\\ r_{21}^{\prime } & r_{22}^{\prime } & \cdots & r_{2n^{2}}^{\prime }\\ \vdots & \vdots & \vdots & \vdots \\ r_{n^{2}1}^{\prime } & r_{n^{2}2}^{\prime } & \cdots & r_{n^{2}n^{2}}^{\prime } \end{array}\right]\left[\begin{array}{c} t_{1}^{\prime }\\ t_{2}^{\prime }\\ \vdots \\ t_{n^{2}}^{\prime } \end{array}\right] \end{array}$$

The solution of the linear equations is the dwell time of APPP to fabricate sinusoidal grid surface. According to the two conditions of the minimum of the residue error and the non-negative dwell time, the optimization objective and constraints of the linear equations are expressed as the follows,

(7) $$\begin{array}{l} \underset{t}{\min}\;g(t)={\left\|R\cdot T-F\right\|}_{2}^{}\\ s.t.{}_{}\;t\geq 0 \end{array}$$

where ${\left\|\cdot \right\|}_{2}$ is the 2-norm of the residual error. The linear equations in Equation (7) can be solved using the non-negative linear square method [17].

## 3. Experiment and Results

### 3.1. APPP of Sinusoidal Grid

**Figure 7:**
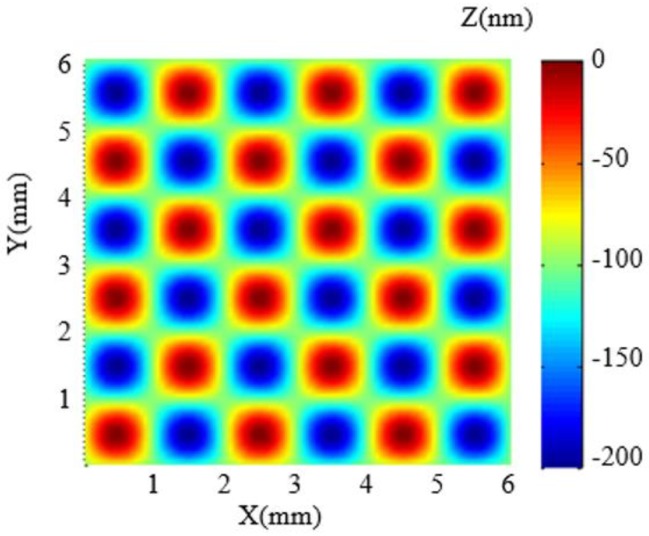
Sinusoidal grid surface for simulation analysis.

**Figure 8:**
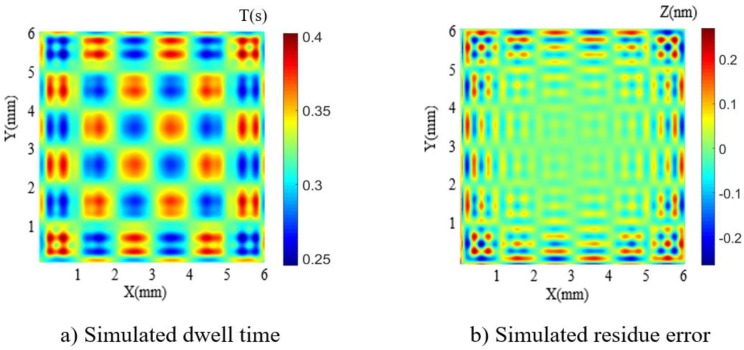
Simulation results.

Before the actual fabrication, simulation is carried out to validate the dwell time solution. In the simulation, the design surface is a 6 mm × 6 mm sinusoidal grid with 100 nm amplitude and 2 mm period in both X–Y direction (as shown in Figure 7) and the selected removal function is experimentally obtained (as shown in Section 2.1). The raster scanning path was adopted and the dwell time was solved using the linear equations method. The computing environment is CPU (Pentium(R) Dual-Core E5300 @2.6GHz) and 3.25 GB memory. The simulation results are shown in Figure 8. It can be seen that the simulated residue error is almost negligible (as the PV and RMS values of the error reach 5.32 × 10^−1^ nm and 6.40 × 10^−2^ nm, respectively), which verifies the dwell time solution of APPP to generate the design sinusoidal grid.

**Figure 9:**
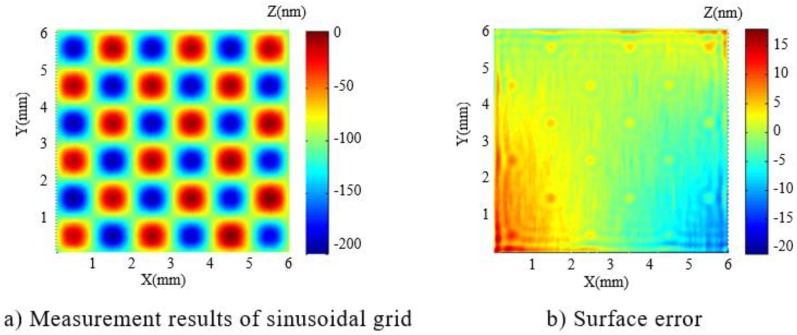
Processing result of sinusoidal grid with period of 2 mm and amplitude of 100 nm.

Based on the simulation results and calculated dwell time, the experimental fabrication was performed using the processing parameters as shown in Table 1. The plasma torch was stabilized for 1 hour beforehand. The fabricated sinusoidal grid surface was then measured by a phase shifting interferometer (Zygo Corp, Middlefield, CT, USA) and the results are shown in Figure 9. The surface error (shown in Figure 9b) shows that the processing error RMS value is 3.2 nm, which indicates the potential of APPP to fabricate optical sinusoidal grid surface with high accuracy.

### 3.2. Post-Processing by Mechanical Polishing

**Figure 10:**
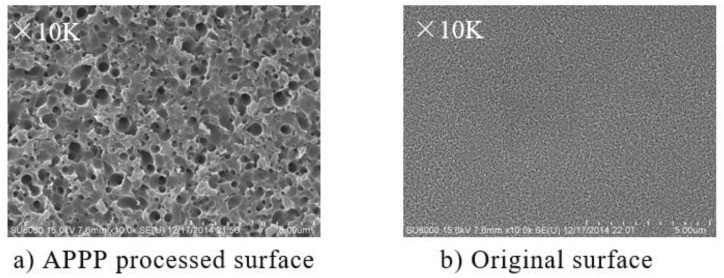
Scanning electron microscope (SEM) images for APPP processed and original surface.

Although the sinusoidal grid surface has been successfully generated, the surface roughness of the fused silica may deteriorate as shown in Figure 10. Due to the chemical etching mechanism of APPP, the surface and subsurface damage evolves to small rough pits, and high density of cellular microstructures are formed, which will increase the surface roughness.

**Figure 11:**
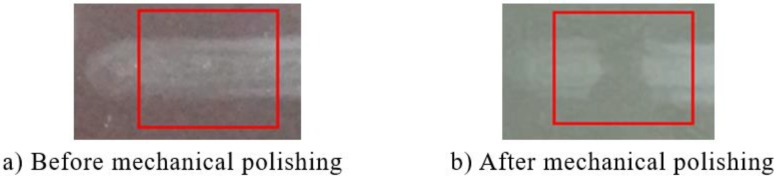
Photographs of APPP processed surface appearance before and after mechanical polishing.

To improve the surface quality, rapid post-polishing based on conventional mechanical polishing method is adopted. A low hardness pitch with 0.2 μm cerium oxide slurry is used to avoid the influence of mechanical polishing on the surface shape and minimize the potential subsurface damage. Figure 11 shows APPP processed surface appearance before and after rapid polishing. The polishing was performed along the vertical direction in the red square.

It can be seen that the roughened area was smoothed and became optically transparent, indicating that the surface roughness was reduced. Atomic force microscopy (AFM) was used for quantitative roughness measurement. After mechanical polishing, the surface roughness reduced from *Ra* 60.0 nm to *Ra* 3.5 nm, which is similar to the surface roughness after SPDT.

In order to evaluate if the post-polishing will generally affect the form accuracy after APPP, a series of grooves (totally 36) with different FWHMs and depths were fabricated by APPP and followed by rapid mechanical polishing. The geometry parameters of grooves are listed in Table 2. The grooves are divided into 3 groups with FWHM of 5, 3 and 1 mm respectively. And each group contains 12 grooves of different depths. Profile measurement (Taylor Hobson PGI 1240) was carried out before and after rapid mechanical polishing.

The influence of the mechanical polishing on the groove profiles was analyzed by calculating the deviation of the groove depths and the FWHMs before and after the mechanical polishing, which is expressed as,

(8) $$Depth\;(FWHM)\;deviation=\frac{Depth\;(FWHM)\;after\;polishing-Depth\;(FWHM)\;before\;polishing}{Depth\;(FWHM)\;before\;polishing}\times 100\%$$

**Figure 12:**
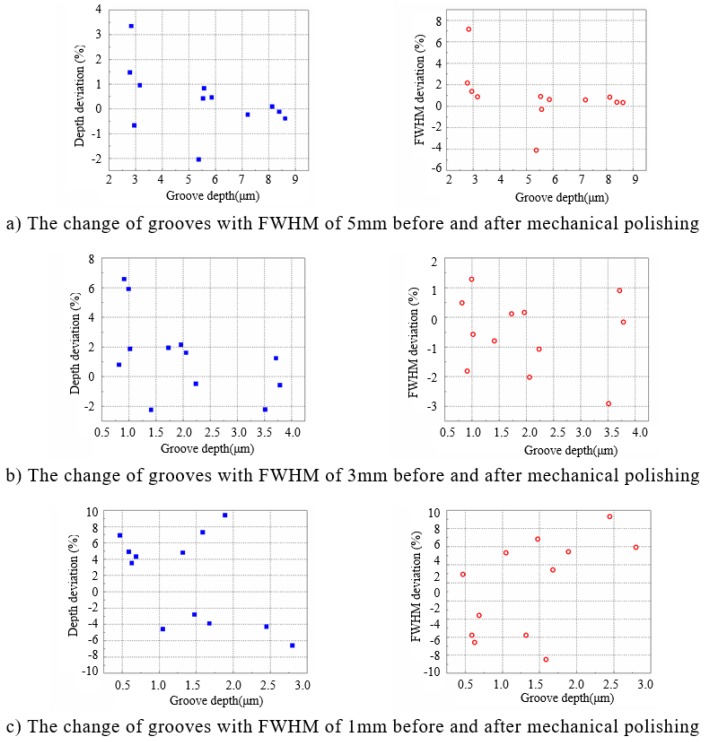
Effects of mechanical polishing on groove profiles with different full width at half maximum (FWHM) and depths.

The results are summarized in Figure 12 and Table 3. When the groove FWHM is 5 and 3 mm, the influence of mechanical polishing on the groove profile is small and the mean deviation value of the groove depth and FWHM is less than 3%; when the FWHM is 1 mm, the effect of mechanical polishing is slightly increased, but the mean deviation value of the groove depth and FWHM is still controlled within 6%. The above results show that APPP features can be well maintained (less than 6% deviation) after rapid mechanical polishing while the roughness is greatly reduced to meet the optical requirement.

**Figure 13:**
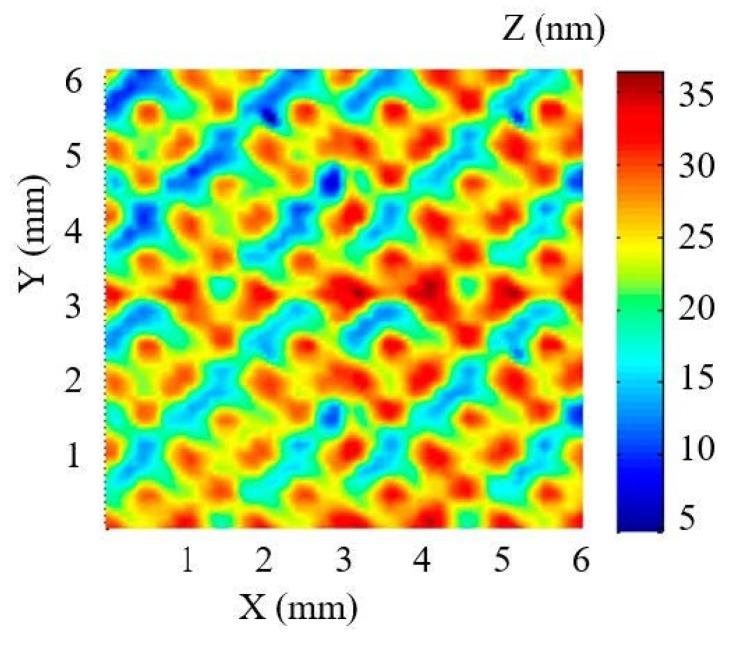
Induced deviation of sinusoidal grid surface by post-polishing.

As for the sinusoidal grid surface fabricated in Section 3.1, the post-polishing was also carried out. The surface form was measured again by the phase shifting interferometer. The difference of two measurements before and after polishing is regarded as the deviation induced by mechanical polishing. As shown in Figure 13, the RMS value of induced deviation is 5.4 nm and acceptable for optical sinusoidal grid.

## 4. Conclusions

This paper has presented the APPP technique to fabricate an optical sinusoidal grid surface. The characteristics of removal function was investigated and linear equations method was adopted to solve the dwell time to generate the design sinusoidal surface. Both simulation and experiment validate the effectiveness of APPP to fabricate a 6 mm × 6 mm sinusoidal grid surface with nanometric precision. Finally, post mechanical polishing was also proposed to reduce the surface roughness after APPP. The groove and sinusoidal grid polishing experiments have shown that APPP features can be well maintained while the roughness is greatly reduced to meet the optical requirement.

## Figures and Tables

**Table 1:** Process parameters of APPP.

He Flow (sccm)	CF_4_ Flow (sccm)	O_2_ Flow (sccm)	Distance (mm)	Power (W)
539	48	5	3	47

**Table 2:** Grooves with different full widths at half maximum (FWHMs) and depths.

FWHM (mm)	Depth (μm)
5	2.8–8.6
3	0.8–3.8
1	0.5–2.8

**Table 3:** Depth and FWHM deviation of grooves after mechanical polishing.

FWHM (mm)	Depth Deviation (%)	Depth Deviation Mean (%)	FWHM Deviation (%)	FWHM Deviation Mean (%)
5	−3–4	0.92	−5–8	1.62
3	−3–7	2.29	−3–2	1.02
1	−7–10	5.28	−8–10	4.94
